# Soldiers' Heart: A Question of Ætiology

**Published:** 1917-05-26

**Authors:** 


### SOLDIERS' HEART.

### A Question of Ætiology.

A recent report by the Medical Research Committee on this subject (Special Report, Series No. 8) is most valuable. A considerable number of cases of "soldiers' heart" was collected in the Hampstead Hospital, which had been lent by the Committee to the Army Council, and a special staff of experienced men was collected to work at them. The advantages were very great. Gathering these numerous cases of "soldiers' heart" from long sojourn in scattered beds elsewhere, and bringing them together in a hospital of exceptional cheerfulness, added nothing to the cost of their hospital treatment; indeed, it very greatly reduced it by shortening its length. It was by the aggregation of the cases itself, moreover, that adequate and coordinated inquiry became possible. By no sacrifice of cost or efficiency, clear light has been gained upon practical problems of immediate military importance, and, it may be added, of general importance to the civil population also.

### The Value of Exercises.

Among the first measures taken was the introduction of a system of exercises for the patients, graded in their severity, and culminating in route marches. One great difficulty that has been encountered by military doctors has been to decide whether and when a patient thus affected should return to duty, and to what class of duty he should return; and there was great difference of opinion as to the symptoms and signs on which reliance should be placed in coming to a decision on this point. The most obvious method does not appear to have been employed until the cases were collected in the Hampstead Hospital. Then the decision was made, not by the existence or absence of any symptom or sign, but by testing the patient as to what he could do—surely a much more satisfactory method. At the same time it was found that the exercises had a very important curative value, so that they served at the same time for treatment and for sorting and classification, and for prognosis.

The special results are to be published in a series of separate articles by the individual workers engaged on the task, but in the meantime certain general conclusions have been attained, and these are published in the monograph before us. The most important of these is that it is practicable by the method of graduated exercises to shorten the average duration of hospital treatment from five or six months to two; and the method has resulted, moreover, in the return to duty of no fewer than 41 per cent. of the cases treated.

Other results of the investigation that stand out prominently as of great importance are that the period of convalescence from infectious disease should be lengthened, so that return to full duty is postponed longer than is the case at present; and that persons taken into the Army from sedentary employments should undergo a more gradual initiation into the laborious physical exertion required. If this were done, many fewer cases of "soldiers' heart" would need to be treated.

Physical signs are found to have little value as indications of the severity of the condition or of the fitness or unfitness for duty. The extension of the apex-beat, even to several rib-spaces, is quite an untrustworthy guide.

It has been clearly shown that digitalis and strophanthus are not beneficial but, on the contrary, tend to make the symptoms worse. Rest in bed is distinctly harmful, and should be avoided at all stages of treatment, except in instances of severe precordial pain, severe headache, or severe giddiness. Continual rest in bed is undesirable under any circumstances. The inclination of many of these patients is to stay in bed; it should be discouraged. Occupation is clearly called for on the ground that it counteracts introspection, which is prevalent. The men should be treated as convalescents; they should be encouraged to stay much in the open air, and should be provided with light employment, such as gardening. They should not only be made to understand that their condition is recoverable, but should be assured from time to time that improvement is manifest. Examination of the heart should not be prolonged, and any suggestion that the heart is affected should be avoided.

### Tobacco and Alcohol.

Other interesting conclusions concern the parts played by tobacco and alcohol respectively in causing this cardiac inefficiency. About 6 per cent. are non-smokers; the most numerous are those who smoke from five to ten cigarettes a day; only 5 per cent. confess to twenty cigarettes or more. About 5 per cent. say that smoking increases their symptoms, and these are, for the most part, moderate smokers. It has been held, but as a matter of sentiment, not of observation, that smoking is the chief cause of the malady. This is disproved by Macgregor's observation that the malady is as frequent among the Sikhs, who are non-smokers, as among other Indian troops. It does seem, however, that smoking aggravates the symptoms.

Of eighty patients, 47 per cent. were lifelong total abstainers, and three more were abstainers until they joined the Service. All the rest but two were very moderate drinkers, and only two out of eighty-three were heavy drinkers. Moreover, the heavy drinkers show a much higher percentage of returns to duty than the abstainers and the temperate drinkers. This result is attributed in the report partly to the fact that the abstainers come mainly from the sedentary class, and partly to the fact that drinking is more frequent in vigorous as opposed to weedy types.

The whole report is of great interest, and leads us to anticipate great value in the more detailed reports that are to follow.

